# Railway Servants' Vision

**Published:** 1892-12-10

**Authors:** 


### The Hospital

An Institutional Journal of

Science, Medicine, Nursing, and Philanthropy.

*For Hospitals, Asylums, Infirmaries, Dispensaries, Medical Practitioners, Students, Nurses, and the Charitable Public.*

Vol. XIII.—No. 324.] [Dec. 10, 1892.

### Railway Servants' Vision.

At the annual meeting of the British Medical Association, held at Bournemouth in 1891, papers on "The Vision of Railway Servants" were read in the section of Ophthalmology by Professor McHardy, of London, and Mr. Beaumont, of Bath, with reference to the English companies, and by Dr. Mackay, of Edinburgh, with reference to the Scottish. Very grave charges were brought against the railway authorities by all those gentlemen; and so important were the issues raised that the Council of the British Medical Association resolved to at once appoint a committee of oculists to inquire into and report upon the whole subject. The committee consisted of the following members: Messrs. N. C. Macnamara, London, Chairman; W. M. Beaumont, Bath; J. H. Bickerton, Liverpool; W. J. Collins, London; G. A. Critchett, London; C. E. Fitzgerald, Dublin; D. Little, Manchester; M. M. McHardy, London; Argyll Robertson, Edinburgh; and G. Mackay, Edinburgh, Hon. Secretary.

The report of this committee has just been issued, and the state of affairs thereby disclosed is in many respects nothing less than scandalous. Assuming, as we may, the strict accuracy of the report, it is not affirming too much to say that, so far as many lines are concerned, if the engine-drivers and guards underwent no sort of visual examination whatever, the danger to the travelling public would not be greater than it is with the ridiculous method of so-called "examination" now in vogue. That the "tests" are utterly absurd and useless all competent persons who read the report will immediately perceive; and yet it is said that the boards of directors, with absolute unanimity refuse even to consider the question of change. No two companies appear to agree either upon the manner of testing or the requisite standard of vision for guards, signalmen, and engine-drivers. For this reckless and criminal condition of affairs no blame can be attached to the district railway surgeons, who, as a matter of fact, are hardly ever consulted about the vision of the employ s. The examination, such as it is, is for the most part conducted by superintendents, inspectors, or clerks; which is the same as setting a jockey to ring the parish bells, or asking a bell-ringer to ride "Ormonde" for the Derby, and to bring him in the winner.

In testing the sight of recruits for the army, it is customary to use the "Dot" method introduced by Professor Longmore, as so many of the men are found to be too illiterate to read the test-types arranged by Snellen. It is not considered necessary for soldiers to have a very high degree of acuity of vision, and the test is, therefore, so arranged that the candidate standing at a distance of fifteen feet can, if he has only one-fourth the normal amount of sight, count black dots of a fixed size on a white background. But a person of perfect vision is able to count the same dots at sixty feet.

Will it be believed that this preposterously-inadequate test is adopted by most railway companies as their normal standard? Some companies are satisfied if a candidate is able to count the dots at the minimum distance of fifteen feet; others demand that he shall be able to read luggage labels at about the same distance; whilst again others expect their employés to recognise the semaphore signals a certain number of yards away. But all such tests are absolutely inadequate for finding out whether men are, or are not, fit for work, in which the question of sight is also a question of life and limb for millions of the public every year.

In the case of colour-vision, the sight of candidates is tested by many of the railway companies by asking the candidate to name the colours of the signals in actual use on the railroad at various distances. Sometimes a sheet of paper of variegated hue is chosen instead of the signals; and sometimes Holmgren's series of worsteds are employed. This latter is, of course, the only test that meets with the approval of the Medical Committee, the others being quite untrustworthy. But, as a matter of fact, any method which depends on *naming* colours is found in practice to be altogether useless as a test of colour-vision.

In addition to a competent first examination of candidates, the re-examination of their vision after the lapse of stated periods of time is considered by the Medical Committee to be absolutely essential for the safety of the trains and the public. In the service of the railway companies at present there appears to be very little agreement as to how long this period should be. In some companies it is not held to be necessary to make any re-examination at all.

That prompt changes are imperatively demanded, both in the direction of uniformity of tests and in their increased stringency, goes without saying. But, that these changes will be made by the companies of their own motion there is no assurance. The probability is that they will not be so much as considered. Railway directors seem to be the last people in the world to adopt improvements until public opinion compels them to advance, whether they will or not.

The report concludes with a list of "recommendations." All those officials who are concerned in the moving of trains, it is recommended, should have their vision carefully examined by competent examiners. Those officials are divided into two classes: Class A consisting of engine cleaners, firemen, drivers, signal-men, and pointsmen; and Class B of all other officials concerned in train moving and signalling. In Class A it is recommended that no candidate shall be passed unless he has quite normal vision in one eye, and not less than half the normal in the other. In Class B selected candidates are to have two thirds of normal vision with both eyes open, provided that neither eye has less than one-third of the standard acuity. In both classes there is to be no restriction of the field of vision, no colour-blindness, no squint, and no disease of the lids, nor of the eyes generally. It is recommended, moreover, with regard to Class A, and it seems almost as important with regard to Class B, that "care should be taken by the examiner to determine that the condition of visual acuteness is not dependent on any latent error of refraction, *e.g.*, high hypermetropia which, by becoming manifest, might lead to the candidates' disqualification in later life." But most errors of refraction, if of much importance, would prevent the candidate reading the types at the requisite distance. Myopia, myopic astigmatism and mixed astigmatism, and hypermetropic astigmatism of moderate degree would thus be revealed; and, as a consequence, the examinee would be promptly rejected. It is probable, however, that now and again a young candidate with a high degree of hypermetropia would, in consequence of his power of accommodation, be able to read the required test-types. How is such an one to be eliminated by a surgeon unpractised in the estimating of errors of refraction? Probably the simplest plan would be to insist that all candidates for service with a railway company shall have their vision tested with the accommodation paralysed by means of atropine. As the candidates are youths, or very young men, there would be no risk of inducing an attack of glaucoma; and the results of the examination, if the instillations were properly carried out, would be unmistakable and absolutely reliable. In this way there would be no difficulty in eliminating the factor of latent hypermetropia, and there would be no necessity for the surgeon to spend time in accurately estimating the exact condition of the refraction. The local surgeons could examine all officials in their own neighbourhoods; and in any case of exceptional uncertainty, the candidate might be sent to an appointed specialist. There is no practical necessity whatever for the centralisation of the work. The ordinary practitioner of to-day is quite capable of acquiring, and in many cases already possesses, a competent knowledge of the technique of vision-testing.

That the railway companies, as we have already hinted, will not readily fall in with any change that may be a source of extra expense is too evident. But the voice of the community is gradually making itself heard in a fashion which even railway directors dare not despise. In the recent accident at Thirsk, the jury, in their verdict, stated that "the persons really responsible for the deaths of the deceased are the directors of the North-Eastern Railway Company." Will the railway companies of the United Kingdom wait for a few coroner's juries to tell them that the farce which they call vision-testing is a standing disgrace and danger to the only civilised country which tolerates such an absurdity?

